# Early-Life Hepatitis E Infection in Pigs: The Importance of Maternally-Derived Antibodies

**DOI:** 10.1371/journal.pone.0105527

**Published:** 2014-08-21

**Authors:** Mathieu Andraud, Maribel Casas, Nicole Pavio, Nicolas Rose

**Affiliations:** 1 Pig Epidemiology and Welfare Unit, Anses, Laboratoire de Ploufragan-Plouzané, Ploufragan, France; 2 Université Européenne de Bretagne, Rennes, France; 3 UMR 1161 Virology, Anses, Laboratoire de Santé Animale, Maisons-Alfort, France; 4 UMR 1161 Virology, INRA, Maisons-Alfort, France; 5 UMR 1161 Virology, Université Paris-Est, Ecole Nationale Vétérinaire d′Alfort, Maisons-Alfort, France; 6 Centre for Research in Environmental Epidemiology (CREAL), Barcelona, Spain; 7 Centre de Recerca en Sanitat Animal (CReSA), Barcelona, Spain; Indian Institute of Science, India

## Abstract

Passive immunity (PI), acquired through colostrum intake, is essential for piglet protection against pathogens. Maternally-derived antibodies (MDAs) can decrease the transmission of pathogens between individuals by reducing shedding from infected animals and/or susceptibility of naïve animals. Only a limited number of studies, however, have been carried out to quantify the level of protection conferred by PI in terms of transmission. In the present study, an original modeling framework was designed to estimate parameters governing the transmission of infectious agents in the presence and absence of PI. This epidemiological model accounts for the distribution of PI duration and two different forces of infection depending on the serological status of animals after colostrum intake. A Bayesian approach (Metropolis-Hastings algorithm) was used for parameter estimation. The impact of PI on hepatitis E virus transmission in piglets was investigated using longitudinal serological data from six pig farms. A strong impact of PI was highlighted, the efficiency of transmission being on average 13 times lower in piglets with maternally-derived antibodies than in fully susceptible animals (range: 5–21). Median infection-free survival ages, based on herd-specific estimates, ranged between 8.7 and 13.8 weeks in all but one herd. Indeed, this herd exhibited a different profile with a relatively low prevalence of infected pigs (50% at slaughter age) despite the similar proportions of passively immune individuals after colostrum intake. These results suggest that the age at HEV infection is not strictly dependent upon the proportion of piglets with PI but is also linked to farm-specific husbandry (mingling of piglets after weaning) and hygiene practices. The original methodology developed here, using population-based longitudinal serological data, was able to demonstrate the relative impact of MDAs on the transmission of infectious agents.

## Introduction

Passive immunity (PI) is the primary protection against infections in early life for several species, including humans [1]–[4]. However, this protection is only partial, rarely totally preventing from infection, but slows down the transmission process among individuals and/or reduces clinical consequences whenever infections occur. Moreover, this protection is only temporary due to maternal antibodies waning and, in the absence of vaccination, individuals become fully susceptible to infection. Although vaccination could overcome this issue, several studies evidenced antagonistic effects between vaccine-induced immunity and maternally derived antibodies through inhibition of vaccine protection, which could potentially worsen the dynamics of infection [5]–[9]. Hence, timely vaccination in regards to passive immunity waning appears crucial to optimize vaccine efficacy.

In this context, we developed a methodological framework to analyze the main characteristics of agent-specific passive immunity, in terms of duration and protection, based on longitudinal data. Our model explicitly takes into account the distribution of passive immunity duration to define the transition rate between passively immune and susceptible states. The protective impact of passive immunity is assessed through the estimation of differential forces of infection (FOI), defined as the *per capita* rate of infection per time unit, in regards with the early life serological status (*i.e.* with or without maternally derived antibodies). A Bayesian approach, based on Monte-Carlo Markov Chains (MCMC), was used to estimate the protection conferred by maternally derived antibodies on the transmission process. Bayesian methodology is of particular interest for the estimations of parameters related to infectious disease dynamics [10]–[18], since such data are subject to uncertainties due, for example, to under-reporting of cases, partially observed processes, or time-aggregated data.

As passive immunity was shown to be partially protective in the context of Hepatitis E virus (HEV) infection in pigs [19], the developed methodology was applied to quantify the protection level conferred to new-born piglets by maternally derived antibodies (MDAs). Hepatitis E virus is a recognized zoonotic agent, which can cause enterically-transmitted hepatitis in humans, and for which domestic pigs are considered as the main reservoir [20]. Understanding the impact of passive immunity on infection dynamics is therefore of pivotal importance to identify strategies to decrease the prevalence of infected animals at slaughter time. To our knowledge, few studies have focused on quantifying the protection conferred by passive immunity (PI) in terms of pathogen transmission in pigs. In 1997, Bouma *et al.* calculated a reproduction number of 0.2 for pseudorabies virus in PI animals (*vs.* 6.3 in absence of MDAs) [5]. Another study on PCV-2 transmission demonstrated a two-fold reduction in pigs with MDAs and an estimated reproduction number of 1.5 [21]. Allerson *et al.* recently studied the transmission potential of swine influenza virus in the presence of homologous and heterologous MDAs (obtained from vaccinated dams) with regard to the challenge strain [22]. The results showed high protection with homologous MDAs but weaker protection in piglets born to sows vaccinated with a heterologous strain. The main risk regarding hepatitis E infection in humans in industrialized countries is the introduction of viremic pigs into the food chain, which is strongly related to the age at infection. Data from a cohort study were first used to characterize MDAs (IgG) kinetics in the early life of piglets (up to 14 weeks of age) and to estimate the distribution of the duration of passive immunity. A second dataset derived from a longitudinal study in 6 Spanish pig-herds [23] was then analyzed, accounting for previous estimates of MDAs waning time, to estimate forces of infection from herd-specific HEV prevalences.

## Materials and Methods

### Distribution of passive immunity duration

A non-linear mixed effect model was used to represent the decay of MDAs titer. The antibody titers *A* in piglets which received maternally-derived antibodies (MDAs) were assumed to decrease exponentially with age according to the equation 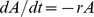. The antibody titers would thus depend on the initial level of antibodies delivered at birth 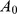 and the antibody decay rate 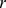. As the initial antibody titer would likely be related to the dam's serological status, dam antibody titers were consequently considered as a covariate related with the initial level of antibodies *A_0_* in offspring. Thus, the model describing serological titer of individual 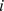 at observation time 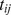 (with a constant residual error model) is given by:



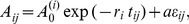



where 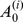 and 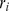 are the individual parameters and 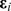
is a vector of standardized random variables. As described in [24], individual parameters were assumed to be log-normally distributed. The serological titer of the dam 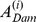
one week after parturition was considered as covariate for the initial serological titer of each individual (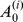). The model for individual parameters is given by:



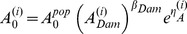
 and 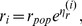



where 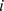 denotes the individual and 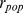 the median decay rate at the population level. 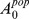 represents the typical initial serological titer in the population, the predicted initial serological titer for individual 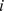 being given by 
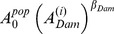
. 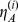 and 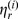 are vectors of random effects assumed as independent centered Gaussian vectors with variance 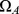 and 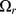, representing inter-individual variability. Parameters were estimated using MLE and the SAEM algorithm for the analysis of hierarchical nonlinear mixed-effects models [25].

Individual antibody kinetics were derived from the individual empirical parameter estimates. Subjects with antibody levels below the cut-off value of the ELISA test were considered seronegative (HEV ELISA 4.0v, MP Diagnostics,Illkirch,France, [26]). Different distributions were tested to model the time to immunity waning (Weibull, lognormal and gamma distributions). Distribution parameters were estimated using the maximum likelihood method and model selection was based on the AIC values. A nonparametric bootstrap procedure was used to determine the 95% confidence intervals for the parameter estimates and thus measure their accuracy. One thousand bootstrap replicates were generated by resampling individual profiles for each dataset. For each bootstrap replicate, selected model was refitted to get an estimate of the distribution parameters. The 95% confidence interval was constructed from the 2.5^th^ and 97.5^th^ percentiles of distribution parameters [27].

### Impact of passive immunity on transmission

PI is known to be a protective factor against infections in early life. However, this protection is rarely fully efficient. We developed a general model framework to represent the evolution of passive immunity with age of the animals. For this purpose, the transition from passively immune to susceptible states was considered as age-dependent according to the estimated distribution described in the previous subsection. The following procedure was adopted: Let 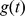 and 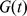 be the probability density and cumulative probability function of this distribution, respectively. Let 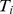 be the interval duration between two observation times 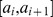.In the absence of infection, animals will then either be passively immune or susceptible and the transition between these two states is governed by:
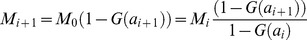
(1)


and 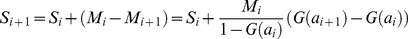
(2)


The second term in equation (2) corresponds to the proportion of individuals losing passive immunity between ages 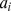 and 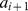. 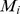 (resp.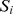) represents the proportion of passively immune (resp. susceptible) animals of age 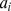.

#### Infectious process

The present study was designed to quantify the protective impact MDAs from longitudinal serological data. For this purpose, and based on equations (1 - 2), the following epidemiological model was developed to account for passive immunity duration and differential forces of infection according to the serological status of individuals at birth.

Let 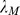 be the force of infection exerted on individuals with passive immunity and 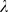 the FOI in the absence of passive immunity (Full model). The probability that an animal with maternal antibodies will escape infection between 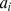 and 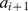 is therefore expressed by 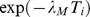 and the proportion of individuals remaining passively immune is given by:
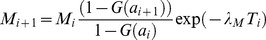
(3)


Individuals for which immunity waning occurs at time 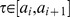 are susceptible at age 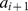 if they were not infected between 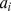 and 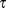 (*i.e.* when they were still passively immune), or between 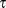 and 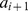, when they were susceptible. Moreover, individuals that were susceptible at age 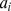 remain so if they escaped infection during the time interval 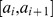 with a probability of 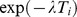. The proportion of susceptible individuals is therefore represented by:
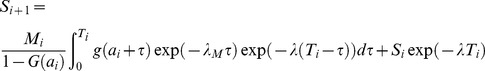
(4)


To clearly identify the impact of PI, we compared this model structure to a simplified version assuming that passive immunity played no role in the transmission process 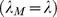. In this sub-model, hereafter referred to as the single force of infection model (SFOI model), the proportion of individuals with passive immunity and susceptible individuals are governed by: 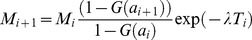
(5)

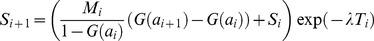
(6)


Whatever the assumption concerning the impact of passive immunity, the proportion of infected individuals 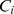 is given by:
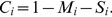
(7)


Model (3-4-7), which considers two forces of infection, is hereafter termed the “full model”, in contrast to model (5-6-7) which considers a single force of infection.

#### Parameter inference

Let 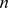
be the number of sampled animals from a total number of 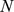
animals per herd. As described by Backer *et al.* [18], the number of infected individuals is not directly observed so the true number of infected individuals of age 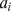 in herd 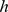, and denoted by 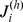 follows the distribution:



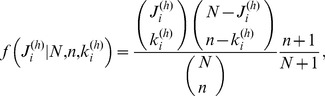
with 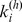 the observed prevalence at age 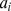 in herd 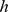.

Considering that the proportion of infected animals at each sampling time is given by equation (7), we can calculate the herd-specific contribution to the likelihood of the observations if there were 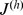 infected animals and 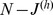 non infected pigs in herd 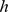:
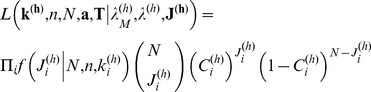



The global likelihood is thus expressed as the product of the herd-specific contributions:



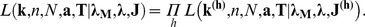
.

A Bayesian framework was used to infer the following parameters: the herd-specific forces of infection 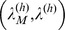, the actual number of infected individuals for each observation time 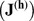 and the initial proportion of passively immune animals (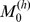). Flat uninformative priors were assumed for all parameters but 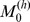 for which a beta distribution was selected in line with the average percentage of seropositive sows in the sampled herds (59%, sd: 10; [23]). Parameters were updated using the Metropolis-Hastings algorithm with gamma proposal distributions for the forces of infection and normal proposal distributions for 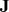 for which the mean value corresponded to the last accepted value and using the variance as tuning parameter. Uniform proposal distributions, also centered on current value (Y ∼ *U*(*M_0;t_ – δ, M_0;t_ + δ*), Y denoting the proposal value and delta being the tuning parameter) were used to estimate 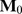. Three chains were run with random initial conditions, 50000 steps per chain, a burn-in of 1000 steps and a thinning parameter of 10. Convergence was assessed by visual inspection and diagnostic tests (autocorrelation plots, Geweke and Gelman-Rubin diagnostics).

### Application to Hepatitis E virus transmission in pigs

No data were specifically collected for the present analysis. Data were available from previous field studies which were described in references [23] and [28].

#### Passive immunity duration

Serological data, used to estimate the duration of PI, were derived from a longitudinal epidemiological study on HEV prevalence in France [28] by monitoring 120 piglets, from 3 consecutive batches in one herd known to be infected by HEV, from birth to 14 weeks of age. Ten sows were chosen at random from this herd and from each followed batch. One week post-farrowing, 4 piglets born to the selected sows were chosen at random and ear-tagged. At the same time, blood samples were collected from the sows to determine their serological status with regard to HEV infection. Blood samples were also taken from piglets at the ages of 1, 6, 10 and 14 weeks for serological analysis.

A commercial test validated for veterinary analyses (HEV ELISA 4.0v (MP Diagnostics, Illkirch, France)) was used to detect anti-HEV antibodies in pigs as described in [26]. This test is based on a double sandwich ELISA that allows the detection of all classes of immunoglobulins (IgG, IgA and IgM) regardless of the animal species. The HEV ELISA 4.0v utilizes a proprietary recombinant antigen, which is highly conserved between different HEV strains. Analyses were performed according to the manufacturer's instructions except that 10 µl of sera were used. Samples were considered positive when the OD450 value of the sample was above the cut-off value (Co  =  mean of the negative control + 0.300).

#### Impact of passive immunity on transmission

The forces of infection in the presence and absence of PI were estimated from data provided by Casas *et al.* [23] because these data had shown evidence of early infections occurring in young piglets in the presence of residual maternal antibodies. Briefly, 6 farrow-to-finish Spanish swine herds of similar size (100–300 productive sows) and known to be infected by HEV were included in the study. In each farm, 20 piglets from 6 previously selected sows, were randomly selected and ear-tagged and serial blood samples were collected at 3, 7, 13, and 18 weeks. Blood was also collected at slaughter (25 weeks of age approximately) at bleeding. We assumed that this number of sampled animals corresponded to 10% of the total number of pigs in the batch (N = 200). In this study, the sera were tested for specific IgG and also IgM which allowed differentiation between passively derived antibodies and a post-infectious response following a recent infection (IgM) [29]. Based on individual serological results, the course of infection was determined from the cumulated number of IgM positive piglets among the 20 piglets examined at each time point.

## Results

### Antibody decay rate

**Figure 1:**
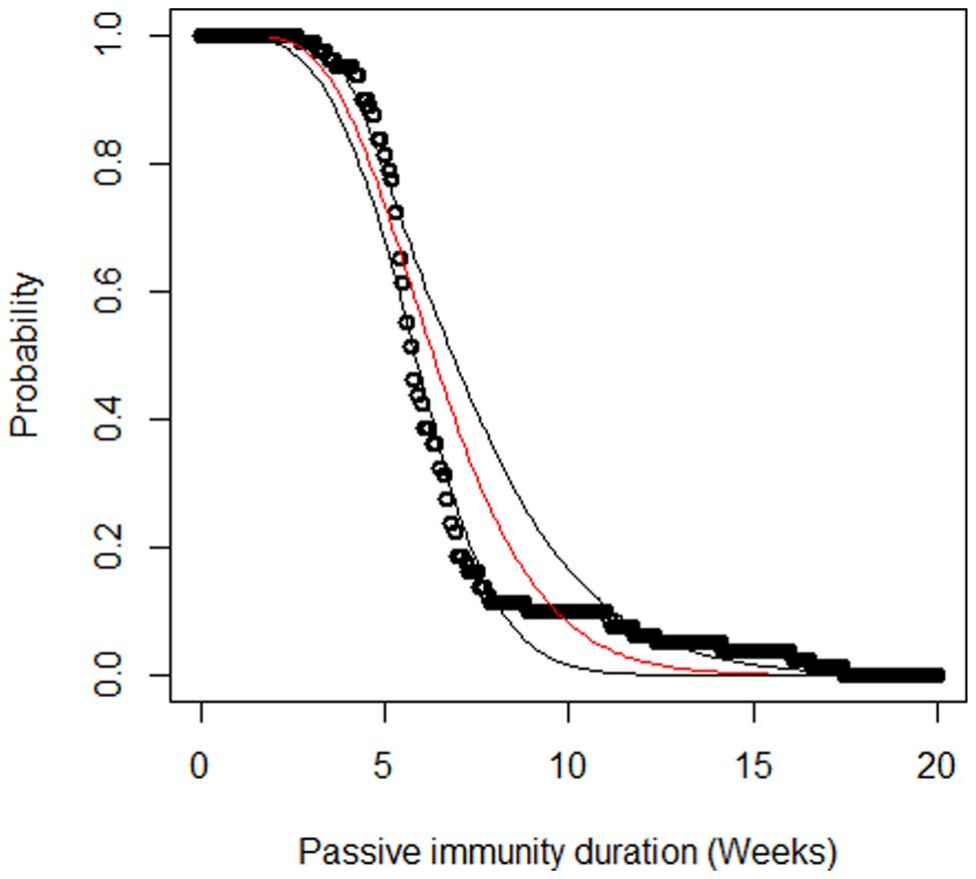
Distribution of the duration of HEV passive immunity (Weeks). We assumed gamma distributions for the duration of PI and used the maximum likelihood method to estimate the shape and scale parameters (red line). 95% confidence bands (black lines) were obtained by the bootstrapping method (see text for details).

The serological investigations in sows revealed a mean HEV seroprevalence of 77% at farrowing with variations (from 70 to 90%) between batches. Seventy percent of the selected piglets were positive for anti-HEV antibodies at one week of age. The MDA titers of piglets were correlated with the titer of their dam (*β_Sow_*  =  0.66, SE = 0.04, p<0.001; Table 1). The estimated antibody decay rate was 0.4 per week (SE: 0.02, Table 1) which corresponded to a half life of 12.1 days. Individual fits of serological profiles are shown in Figure S1. The individual duration of PI, based on the individual parameter estimates, was determined by long term projection of the antibody kinetics (assuming a threshold value of 0.38 Optic Density (OD)). Different distributions were fitted to the individual durations of PI (Weibull, lognormal and gamma distributions). The mean durations of PI were similar for all distributions but the gamma distribution was selected, based on the Akaike Information Criterion (AIC). Thus, the duration of PI was fitted to a gamma distribution with a mean duration of 6.5 weeks (95% CI: [6.0; 7.2]; Figure 1). This distribution was then used to model the transition between the PI state and the fully susceptible state in the epidemiological model (Equations (3–7)).

**Table 1:** Parameter estimates of HEV maternal antibodies kinetics.

	Parameter estimate	SE	p-value
*A_0_*	0.47	0.07	
*r*	0.4	0.02	
*β_Dam_*	0.66	0.04	<10^−3^

*A_0_*: initial antibodies concentration (ELISA test Optic density (OD)).

*r*: antibody decay rate (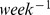).

*β_Sow_*: sow's serological titer covariate coefficient on piglets' initial antibody titer.

### HEV Infectious process and passive immunity

**Figure 2:**
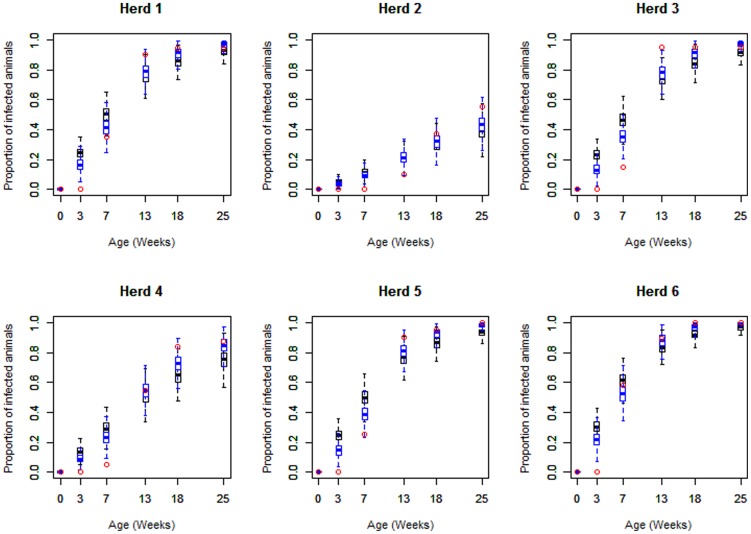
Estimated age-specific HEV seroprevalences. The distributions of estimated seroprevalences are shown according to model structure (SFOI model: black boxplots; Full model: blue boxplots). Observed prevalence data, derived from the longitudinal data in six pig herds in Spain [23], are represented by red dots.

Convergence of the MCMC Algorithm is shown in figure S2. A graphical representation of the posterior distribution of herd-specific prevalences, *i.e.* the proportion of infected individuals (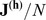), according to the model structure is provided in Figure 2. As previously highlighted by Casas *et al*. [23], all but one herd (herd 2) exhibited similar profiles with a huge increase in prevalence between 7 and 13 weeks of age, leading to asymptotic behavior close to 1. This increase in prevalence coincided with the estimated duration of PI, suggesting an impact on transmission dynamics. Visually, the estimated prevalence values based on the full model (blue boxplots) were better adapted to reproduce this behavior than the SFOI approach (black boxplots).

The force of infection estimates, obtained by using the SFOI model, ranged between 0.019 and 0.136 week^−1^ (Table 2). They tended to overestimate the predicted prevalence of infected animals before 7 weeks of age and led to underestimations thereafter. The full model structure was able to disentangle the transmission process in the presence and absence of PI. Both visual inspection (Figure 2) and the DIC values (Table 2) led us to conclude that the model with two different forces of infection better captured the dynamics of infection observed in field conditions. With this full model, the forces of infection exerted on fully susceptible individuals were estimated on average 1.8 times higher than with the SFOI model (range: 1.3 – 2.0; Table 2). In contrast, transmission in piglets with PI was found, on average, 13 times lower than in fully susceptible animals, with a relatively large variability between herds (range: 5–21).

**Table 2:** Estimates of forces of infection according to piglets' serological status.

	Herd N°	λ^1^	λ_M_ 2	DIC
**SFOI model** 3	1	0.105 (0.079;0.140)	–	90
	2	0.019 (0.012;0.028)	–	81
	3	0.099 (0.074;0.129)	–	101
	4	0.055 (0.038;0.077)	–	94
	5	0.106 (0.079;0.140)	–	94
	6	0.136 (0.102;0.181)	–	88
**Full model** 4	1	0.181 (0.109;0.290)	0.017 (9E-04;0.082)	78
	2	0.025 (0.015;0.042)	0.006 (5E-04;0.032)	78
	3	0.190 (0.122;0.301)	0.009 (7E-04;0.048)	83
	4	0.092 (0.056;0.140)	0.008 (7E-04;0.042)	84
	5	0.215 (0.129;0.351)	0.010 (5E-04;0.054)	75
	6	0.274 (0.148;0.615)	0.023 (0.001;0.112)	75

1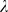: Posterior median estimate with 95% credibility interval of the force of infection without PI (week^−1^).

2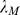: Posterior median estimate with 95% credibility interval of the force of infection in the presence of PI (week^−1^).

3 SFOI: Single force of infection model (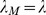).

4 Full model: differential forces of infection in the presence and absence of MDAs (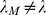).

**Figure 3:**
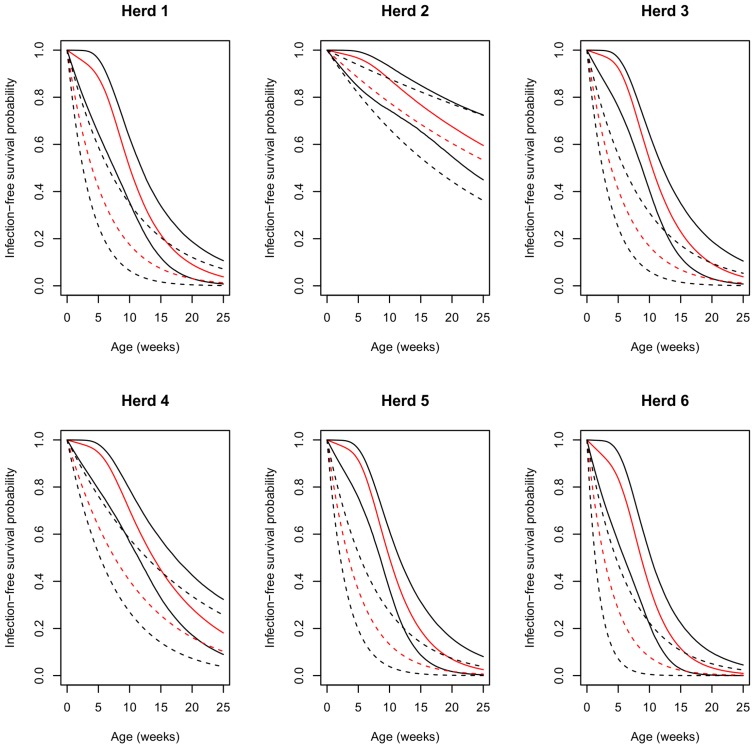
HEV Infection-free survival function. Median (red curves) and 95% credibility interval (black curves) of age specific probability to escape infection according to piglet's serological status after colostrum intake (dashed lines: fully susceptible animal; full lines: pig with HEV passive immunity).

The impact of PI on time to infection was assessed by modeling the infection-free survival functions in piglets with MDAs (Figure 3; full line) and fully susceptible animals (Figure 3; dashed lines) for each herd. In the herds with high prevalence (all but herd 2), fully susceptible individuals escaped infection according to exponential distributions with median infection-free survival ages ranging between 2.7 and 7.6 weeks. In contrast, in passively immune animals, the combined gamma-distributed duration of PI and the differential forces of infection produced sigmoid survival curves with a slight probability of infection in the presence of MDAs (before 7 weeks of age), followed by an increased probability of infection thereafter. In consequence, the infection-free survival age in piglets which had received maternal antibodies was much higher than in fully susceptible animals, ranging between 8.7 and 13.8 weeks. In herd 2, the infection-free survival curves were similar in shape for both fully susceptible and passively immune animals (median: 27.5, 95% CI: [17.0; 53.4] and 32.0 weeks, 95% CI: [22.3; 51.5], respectively), but are not shown in Figure 3 since they extended beyond the normal slaughter age.

## Discussion

Piglets do not receive any maternal antibodies during gestation due to the epitheliochorial placentation in sows [30]. Neonate piglets are thus susceptible to the majority of infectious agents to which they are exposed in early life. It is therefore essential to provide new-born piglets with MDAs via colostrum to protect them against viral and bacterial infections before their own immune system develops [31]. However, the protection conferred by PI has been shown to be partial for several pathogens, reducing the clinical expression and/or within–host viral replication in infected piglets [21], [32]–[35]. To our knowledge, the extent to which MDAs affect the transmission process has rarely been quantified to date [5], [21], [22]. We present an original approach to investigate and model the impact of PI on the transmission of an infectious agent in pigs based on batch-level prevalence data using two nested modeling structures: (i) differential forces of infection dependent on the serological status of piglets following colostrum intake (full model); and (ii) an equal force of infection exerted on fully susceptible and passively immune animals (SFOI model). This modeling approach, which combines a realistic distribution of the duration of PI and differential forces of infection, with and without maternal antibodies, provides a better understanding of early-life transmission of infectious agents. This approach was used to analyze serological data from a longitudinal study on hepatitis E prevalence in 6 pig herds.

The model only provides a simplified representation of the actual process of hepatitis E virus transmission with a main shortcut relying on the assumption of constant forces of infection. Indeed, the role of the environment on the course of infection was highlighted in a previous study, indicating that the force of infection represents a combination of transmission by direct contact between animals and ingestion of viral particles present in the environment [36]. Moreover, it is likely that the susceptibility to infectious agent in passively immune individuals varies as the level of maternally derived antibodies decreases with age. Thus, the estimated FOIs should be considered as the average infection pressure exerted on individuals to assess and quantify the protection conferred by PI. As all pigs within a specific herd were reared under the same conditions, the transmission process was summarized by forces of infection, by considering that a similar infection pressure was exerted on each individual independently of their serological status after colostrum intake. In consequence, the protection induced by PI is clearly related to a decrease in susceptibility.

The aim of the present study was to quantify the protective impact conferred by PI against Hepatitis E infection. Data from a longitudinal survey in France were used to characterize the kinetics of maternally-derived antibodies in 120 piglets. The serological titers of piglets at 1 week of age were highly correlated with those of the dam, thus supporting previous observations [37], [38]. The duration of PI was modeled by a gamma distribution with a mean duration of 45.6 days [41.4; 50.4]. Although the duration was slightly shorter than in several epidemiological studies (8–9 weeks of age) [19], [37], [38], because of the gamma distribution, the probability of remaining passively immune fell below 0.1 by 60 days of age, as in these previous studies. In a recent study, the transmission parameters for hepatitis E virus in pigs were estimated from field data and the effectiveness of different hypothetical control strategies (early life vaccination and delayed vaccination around 10 weeks of age) was evaluated [18]. The author concluded that vaccination should lead to a 75% reduction of the main parameters governing the transmission process (host susceptibility, transmission rate or infectious period duration) to significantly decrease the expected prevalence at slaughter age. However, several studies showed interference between the maternally-derived antibodies and the immune response after vaccination [5], [6], [32], [39], [40]. Such interactions could have dramatic consequences on transmission. For example, the study by Bouma *et al.* revealed an increased reproduction number in piglets vaccinated against pseudorabies virus in the presence of PI as compared to vaccinated piglets without MDAs [5]. Further work should therefore be carried out to assess possible interactions between MDAs and future candidate vaccines before considering their usefulness in HEV control. However, a delayed vaccination strategy, as studied in [18], would certainly avoid such interactions as only 10% of piglets who received MDAs remained passively immune at 10 weeks of age.

Based on individual serological data for 120 pigs from 6 herds published by Casas *et al.* [23], we estimated the herd specific cumulative incidence by assuming that the first-positive IgM detection reflected a recent infection. Two nested model structures were tested assuming either an equal force of infection in the presence and absence of MDAs (SFOI) or two different forces of infection (full model). However, neither model fitted the null prevalence observed at 3 weeks of age for two main reasons: (i) the estimated observed initial proportion of passively immune animals was about 60% in all herds so that 40% of the animals were deemed fully susceptible at birth; (ii) even in the presence of PI, piglets are not fully protected against infection as shown by the non negligible FOIs estimated in the presence of maternal antibodies. Moreover, only 20 piglets in each herd were sampled to establish the herd-specific seroprevalence profiles, which might have led to considerable uncertainty, especially if prevalence was low. These considerations are supported by the good concordance between the estimates and the data from 7 weeks of age onwards. In herd 2, the prevalence increased slowly, continuously and exponentially. Indeed the cumulative prevalence observed at slaughter age was about 50%. As the level of PI was similar to that of the other herds, this difference reflects a low infection pressure which might be due to differences in husbandry practices and herd structure.

In models with a single force of infection, the inverse of the force of infection corresponds to the average age at infection [41]. However, this relationship cannot be applied to the full model due to the interplay between piglet serological status and the differential forces of infection. The impact of PI on time-to-infection was therefore assessed by modeling the infection-free survival functions, *i.e.* the probability of escaping infection according to age of the animal, in piglets with and without MDAs, for each herd. In a fully susceptible animal, transmission occurs much earlier than in pigs with MDAs. Several epidemiological studies have shown that peak HEV prevalence occurs after the nursery stage, at about 13 weeks of age, which corresponds to the estimated median age at infection in passively immune individuals [19], [38], [42]. However, the predicted average age-at-infection in herd 2 was considerably higher and beyond the usual slaughter age (25 weeks). As the level of PI in all herds was similar, this difference could presumably only be explained by differences in herd structures and husbandry practices which might help to reduce the infection pressure due to piglet mixing and the environmental viral load. These results suggest that the age at HEV infection does not strictly depend on the proportion of passive immune piglets but also relies on later events which condition the force of infection after PI waning [43].

The origins of most autochthonous HEV cases remain undetermined, although some cases have been related to the consumption of uncooked meat, liver or offal from domestic pigs or wild animals (*e.g.* wild boar or deer) [44]–[46]. In consequence, control measures in pig farms are aimed at preventing the presence of viremic animals at slaughter age. The modeling framework developed in this study was successfully used to evaluate the age at infection according to the serological status of piglets after colostrum intake in different pig farms. In herd 2, a large proportion of pigs remained susceptible before slaughter-age, mainly due to a lower force of infection after the wane of PI. These conditions could be associated with an increased risk as regards public health especially if later events, shortly before slaughter, enhance the infectious process and lead to massive infection of pigs. Further work needs to be carried out to fully understand which factors are involved in the resulting variations in forces of infection between herds.

We developed an original modeling framework to analyze the role of passive immunity in the transmission of infectious agents in animal populations based on batch-level incidence data. The protective characteristics of MDAs, acquired either through colostrum intake or transplacental passage, play an important part in several mammalian species [47]–[49]. In consequence, the methodology developed in this study could be extended to quantify the level of protection conferred by maternally-derived antibodies for different pathogens and species.

## Supporting Information

Figure S1 **Individual fits of decay of maternally derived antibodies (12 individual profiles are shown).**
(TIFF)

Figure S2 **Convergence plot of Monte Carlo Makov Chains for Herd 1 parameters (lines correspond to smoothing averages for each chain).**
(TIFF)
