# Supplementary figures and images for: Early-Life Hepatitis E Infection in Pigs: The Importance of Maternally-Derived Antibodies
Source: PLoS One. 2014 Aug 21;9(8):e105527. doi: 10.1371/journal.pone.0105527 (PMC4140806; doi:10.1371/journal.pone.0105527)

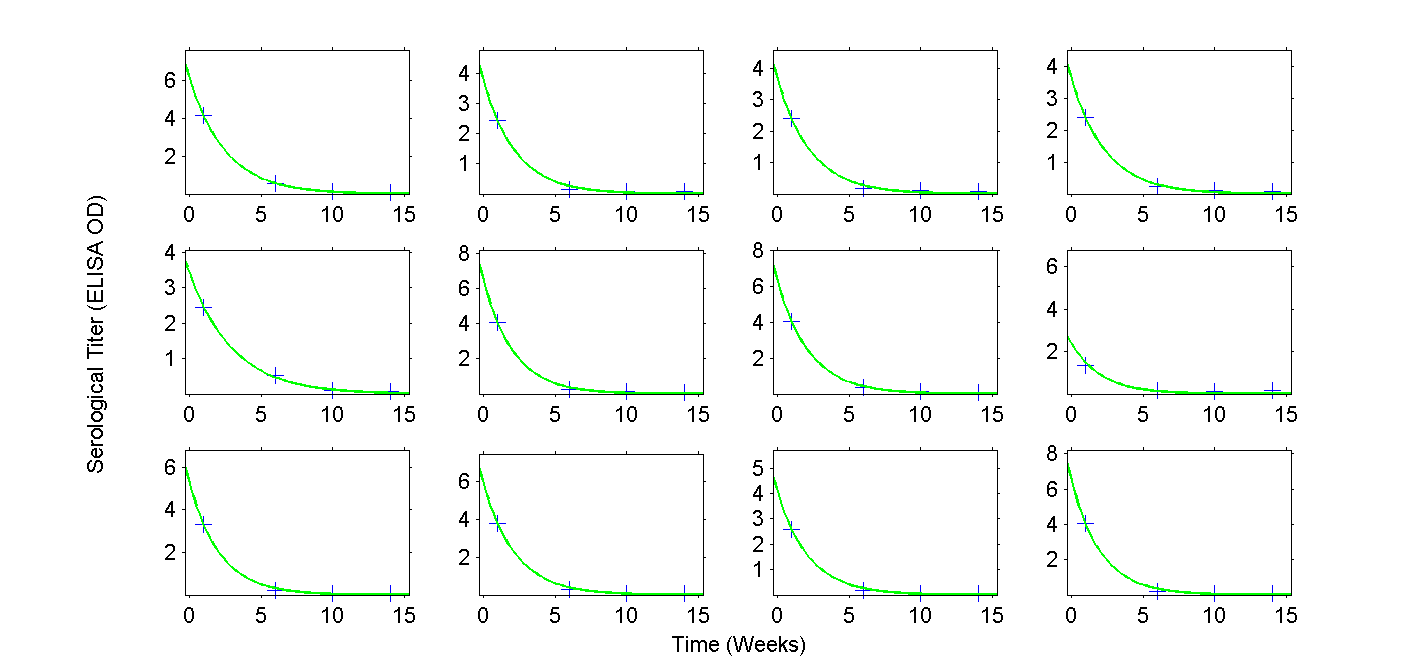

Supplement: Figure S1 — Individual fits of decay of maternally derived antibodies (12 individual profiles are shown). (TIFF) [file pone.0105527.s001.tiff]

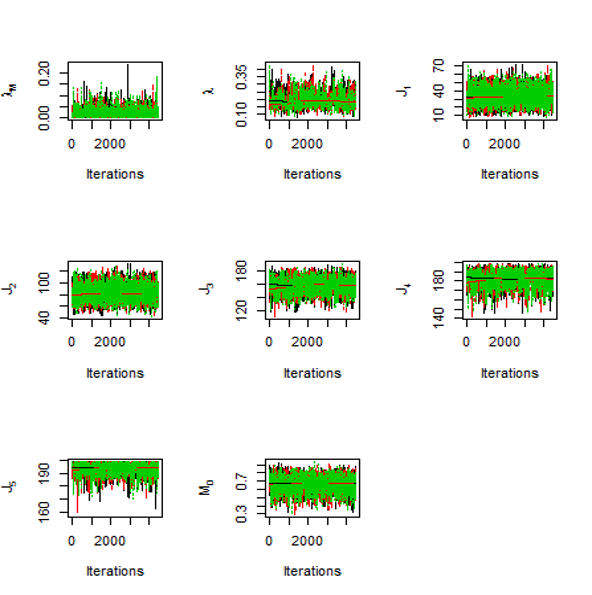

Supplement: Figure S2 — Convergence plot of Monte Carlo Makov Chains for Herd 1 parameters (lines correspond to smoothing averages for each chain). (TIFF) [file pone.0105527.s002.tiff]
